# Under five diarrhea among model household and non model households in Hawassa, South Ethiopia: a comparative cross-sectional community based survey

**DOI:** 10.1186/1471-2458-14-187

**Published:** 2014-02-20

**Authors:** Fekadeselassie Berhe, Yemane Berhane

**Affiliations:** 1 Hawassa University College of Medicine and Health Science, Hawassa, Ethiopia; 2 Addis Continental Institute of Public Health, Addis Ababa, Ethiopia

**Keywords:** Under five diarrhea, Model household, Health service extension program, Hawassa

## Abstract

**Background:**

Ethiopia has been implementing a community-level health intervention package (referred to as “Health Extension Program”) to improve the health of children in particular.

However, its effect on the major childhood illnesses in Ethiopia has not been studied. This study was conducted to determine whether a fully-implemented health extension program reduces diarrhea in children under the age of five.

**Method:**

A Community-based comparative cross-sectional study was carried out by comparing model households (i.e. households that fully implemented the health extension package) with non-model households (i.e. households that did not fully implement the health extension package). The study participants were mothers having children under the age of five. Data were collected through a household survey. A multiple logistic regression model was used to control known confounders.

**Result:**

After controlling potential confounding factors using a logistic regression model, under five year children residing in non-model households, were more likely to have diarrhea in the two weeks preceding the survey compared to those residing in model households [AOR: 2.65, 95%CI (1.11, 6.27)].

**Conclusion:**

Diarrhea among under five children significantly reduced among families who fully implemented basic health packages. The finding suggests that being a model HH can have a positive impact on diarrhea morbidity among under five children.

## Background

Ethiopian health sector policy focuses on prevention of major communicable diseases. In order to control such diseases, and to deliver primary health care (PHC) in the rural communities, the government implemented various strategies in the last two decades. The Health Sector Development Program I (HSDP), (1997/98-2001/02) demonstrated the government’s policy of putting disease prevention at the center of its reorganization of the health service delivery system. The main priorities were to expand and rehabilitate PHC units, improve and expand district hospitals and promote equity by focusing on rural parts of the country. But the review of HSDP I indicated that, there were challenges in universal coverage of PHC and revealed that basic Health services are not delivered to the people at the grass root level. In response to this challenge, and to strengthen preventive, promotive and basic health care at household (HH) level, the government introduced community–level intervention called health service extension package (HSEP). The goal of the HSEP is to improve access and equity in health care and to bring positive behavioral change towards the maintenance of a healthy environment through the provision of house to house health awareness with active community participation. It is the main pillar of the Child Survival Strategy for increasing access to promotive, preventive and some basic essential curative health services to the majority of the underserved population [1,2].

The Health services extension package includes interventions under four main categories including: Family Health Services (Maternal and child health, Family planning, Immunization, Adolescent reproductive health and Nutrition), Infectious disease Prevention and Control (TB, HIV/AIDS, STI, and Malaria), Hygiene and Environmental Sanitation (Excreta, Solid and liquid waste disposal, Water supply, Food hygiene, Housing, Personal hygiene, Vector and rodent Control) and Health Education and Communication. The HESP is implemented by full-time female health extension workers in the community. They train selected households, which are pro-change and influential community members to implement the packages fully. Those households that successfully implement all four components are labeled as “model households” and they are officially certified [3]. Different studies conducted after the implementation of this HSEP indicated that, there were improvements in the community health. Some of the improvements were; latrine construction and utilization, awareness and knowledge on hand hygiene, community awareness on different health issues, increase in immunization coverage, maternal service utilization and balanced diet preparation [4-10]. The health status of model households which implement the HSEP fully is assumed to be superior to non-model households. Therefore, the objective of this study was to assess the effect of full implementation of the health extension package at a household level on childhood diarrhea by comparing model and non-model households.

## Methods

The study utilized a comparative cross-sectional community-based survey design. It was conducted in Tula sub city which is one of the sub cities found in Hawassa, a city located 275 kilometers away from the capital city Addis Ababa. Out of eight sub cities in Hawassa, Tula was purposely selected since it is the only sub city having model HH training. In this sub city, there are 11 rural kebeles (small administrative unit) with 18272 children under five years old. According to the sub city health department report, model HH training was started in 2007/2008 GC. From the total of 26061 HHs found in the administration, 13559(52%) received model HH training up to 2011 GC. In the area around 90% of the residents are farmers and health services are provided by two health centers and 11 health posts.

A multistage cluster sampling technique was applied; sampling was done at the kebele, “gott” and household levels. In the first stage, 3 out of 11 rural kebeles were randomly selected. In these 3 kebeles, there are 60 neighborhoods (locally referred to as “gotts”). Out of these 60, 12 “gotts” were randomly selected. Then, from each selected “gott” households with children under 5 years of age were selected. The total sample size needed for the study was calculated with the aim of detecting difference between model and non model households of 12.5% and 25.0%, respectively with a power of 90% and confidence level of 95%. Then we added for non-response and design effect of 2. Accordingly 434 under five children from model and 434 from non model HHs were needed for the study. Under five year children residing in model and non model households were identified through a house to house enumeration prior to the actual data collection.

The actual data collection was carried out in January 2012 by trained nurses using a structured questionnaire administered to the mothers of the under five children in the selected households. The main outcome variable was having diarrhea in the two weeks prior to the interview. Socio economic, demographic factors, housing, sanitation related hygiene behaviors and water handling characters were among the variables collected.

In order to prevent interviewer bias during data collection, households were coded and data collectors were blinded regarding whether the household was model or non model. All field workers were trained prior to data collection and regular supervision was done during the field work. Each data collector checked the questionnaires for completeness before leaving each study participant. All filled questionnaires were reviewed at the end of the day by the supervisor. Each questionnaire was given a unique code and data were entered using EPI-DATA. Frequencies and proportions were used for description of the study population. SPSS version 16 was used for data analysis. Adjusted odds ratios with 95% confidence interval were calculated using a logistic regression model to control for known confounding factors.

The study was approved by the Ethical Review Committee of Hawassa University College of Medicine and Health Science and Hawassa City Administration granted permission to conduct the study. During data collection individuals were informed about the purpose of the study, confidentiality, and the right not to participate or withdraw at anytime. Children that were found sick during the study period were referred to the nearby health institution or health post for further management.

## Results

A total of 866 children under the age of five years residing in 650 HHs were enrolled in the study. Of these children, 432 were residing in 327 model HHs where as 434 were residing in 323 non model HHs. The response rate for the study was 99% for model and 100% for non model HHs. The mean HHs size of the study population was 5.91 (± 1.85 SD) persons in model and 5.84 (± 1.92 SD) in non model HHs. The majority of the caregivers, 243 (74%) and 240 (74%) had no formal education and, 270 (83%) and 246 (76%) had livestock in model and non model HH respectively. Concerning environmental and hygiene related characters, no statistical difference was observed between model and non model HHs, such as; distance to latrine (P-value 0.70), availability of hand washing facility around the latrine(P-value 0.20), drinking water handling practices (P-value 0.59), and hand washing practice before feeding a child(P-value 0.29).

On the other hand, Tables 1 and 2 show that there were statistical differences observed between the two groups in latrine availability, number of rooms, living with domestic animals in the same house, feces seen around the house, childhood diarrhea, not washing hand after toilet visit, and disposal method of child feces in a latrine.

**Table 1:** Socio demographic, economic and Environmental condition of model and non mode HHs, Tula sub city, Southern Ethiopia, January 2012

**Variables**	**Model HHs(327)**		**Non Model HHs(323)**			
	**No**	**%**	**No**	**%**	**X**^**2**^**Test**	**P value**
Family size						
<5	150	45.9	161	49.8	1.03	0.31
>5	177	54.1	162	50.2		
Number of under 5 children in households						
1.	229	70	216	66.9		
2.	95	29.9	104	32.2	0.76	0.68
3.	3	0.9	3	0.9		
Maternal age						
15-24	52	15.9	65	20.1		
25-34	202	61.8	202	62.5	3.66	0.16
35-49	73	22.3	56	17.3		
Maternal education						
No formal education	243	74.3	240	74.3		
1-6	70	21.4	68	21.4	0.06	0.97
≥7	14	4.3	15	4.6		
Cash crop						
Coffee	51	15.6	57	17.6		
Chat	62	19	70	21.7	1.61	0.06
Both	112	34.3	101	31.3		
None	102	31.2	95	29.4		
Latrine availability to use						
Yes	301	92	237	73.4	39.73	0.001
No	26	8	86	26.6		
Number of room						
1 room	66	20.2	97	30		
2 rooms	162	49.5	161	49.8	15.63	0.001
≥3 rooms	99	30.3	65	20.2		
Animal live with people (n = 516)						
Yes	196	72.6	209	85	11.66	0.001
No	74	27.4	37	15		
Hand washing facility around the latrine						
Yes	32	11.1	17	7.7	1.68	0.19
No	256	88.9	204	92.3		
Feces seen around the house						
Yes	57	17.4	91	28.2	10.66	0.001
No	270	82.6	232	71.8		

**Table 2:** Maternal child care giving and hygiene related behavior characteristics of model and non model household, Tula sub city, Southern Ethiopia, January 2012

**Variables**	**Model HHs(327)**		**Non Model HHs(323)**			
	**No**	**%**	**No**	**%**	**X**^**2**^**Test**	**P value**
Current breast feeding status (n = 52)						
Exclusive	21	87.5	22	78.6	0.72	0.39
Partial	3	12.5	6	21.4		
Time of introducing supplementary feeding (n = 814)						
Not started at 6 month	2	0.5	2	0.5		
< 5 month	11	2.7	11	2.7	1.99	0.57
6-9 month	393	96.3	393	96.8		
> 10 month	2	0.5	0	0		
Diarrhea in the last two weeks (n = 866)						
Yes	40	9.3	61	14.1	4.83	0.03
No	392	90.7	373	85.9		
Vitamin A supplementation (814)						
Yes	402	98.5	403	99.3	0.99	0.32
No	6	1.5	3	0.7		
Water collection container Cover						
Yes	299	91.4	287	88.9	1.22	0.27
No	28	8.6	36	11.1		
Water storage container cover (n = 515)						
Yes	274	91.8	229	93.1	0.29	0.59
No	22	8.2	17	6.9		
Hand washing after toilet visit						
Yes always	39	11.9	13	4		
Yes some times	102	55.7	165	51.1	19.87	0.001
Not at all	106	32.4	145	44.9		
Disposal method of child feces						
Child always use latrine	4	1.2	4	1.2		
Child faces are always thrown in to latrine	235	71.9	191	59.1	12.42	0.006
Child faces are buried in yard	28	8.6	35	10.8		
Child faces thrown outside	60	18.3	93	28.8		
History of diarrhea in past 2 week of mother						
Yes	19	5.8	20	6.2	0.04	0.84
No	308	94.2	303	93.8		

Of the total under five children, 232 (54%) and 232(55%) were male in model and non model HHs respectively. The mean age of children was 32.25(±15.9 SD) months in model and 31.56(±16.35 SD) months in non models. Two hundred twenty nine (70%) in model and two hundred sixteen (67%) in non model HHs had one under five child. Of those children greater than 6 months, vitamin A supplementation was received by 402 (99%) in model and 403 (99%) in non model HHs. Out of 24 under 6 month children, 21(88%) in model HHs, while out of 28, 22(79%) in non model HHs mothers’ breast feed their children exclusively. Almost 97% caretakers started supplementary feeding in the age between 6-9 months in both groups. Concerning diarrheal morbidity history, 40(9%) in model and 61 (14%), in non model HHs had diarrhea in under five children in the past two weeks prior to the survey. In model and non model HHs’ child, diarrhea lasted for less than 14 days and children between age one and three years old were more affected.

After controlling potential confounders using logistic regression a statistically significant diarrheal disease occurrence was observed between the two groups. Children from non model HHs were 2.6 times more likely to have diarrhea than children who are from model HHs [OR: 2.65, 95%CI (1.11, 6.27)]. Other risk factors related to under five diarrhea were; maternal diarrheal morbidity [OR:3.73,95%CI(1.08,12.89)], covering drinking water collection container [OR:0.28,95%CI(0.10,0.79)], covering drinking water storage container [OR:0.12,95%CI(0.04,0.39)] and maternal education [OR: 0.23, 95%CI (0.06, 0.87)] as shown in Table 3.

**Table 3:** Variables significantly associated with childhood diarrhea morbidity, Tula sub city, January 2012

**Independent variable**	**Childhood diarrhea**		**Crude OR (95%CI)**	**Adjusted OR (95%CI)**
	**Yes (%)**	**No (%)**		
Household type				
Model	40(9.3)	392(90.7)	1.00	1.00
Non model	61(14.1)	373(85.9)	1.60(1.05,2.45)*	2.65(1.11,6.27)*
Drinking water collection container have cover				
Yes	53(8.5)	573(91.5)	0.19(0.10,0.36)**	0.12(0.04,0.39)**
No	22(25.0)	66(75.0)	1.00	1.00
Drinking water storage container have cover				
Yes	53(8.5)	573(91.5)	0.19(0.10,0.36)**	0.12(0.04,0.39)**
No	17(32.7)	35(67.3)	1.00	1.00
Maternal diarrhea morbidity				
Yes	17(34.7)	32(65.3)	4.64(2.47,8.71)**	3.73(1.08,12.89)*
No	84(10.3)	733(89.7)	1.00	1.00
Maternal education				
No formal education	84(13.2)	55(86.8)	1.00	1.00
1-6	14(7.2)	181(92.8)	0.51(0.28, 0.91)*	0.23(0.06, 0.87)*
≥7	3(8.1)	34(91.9)	0.58(0.17, 1.92)	0.43(0.05, 3.68)

**Variables entered**: household type, education of mother, occupation of father, number of rooms, refuse disposal method, animals in the living room, separate kitchen, same container for collection and storage, cover of storage and collection container, hand washing habit after toilet visit, availability of soap, maternal diarrhea, age of child and latrine.

**N.B** *indicate significance at P < 0.05, **indicate significance at P <0.001.

## Discussion

This study shows that, there is a significantly higher prevalence of diarrhea among children residing in non model households compared to those residing in model households. This difference may be due to the fact that, out of three implementation components of HSEP (provision of community based health package, capacity building of potential families to be role model HHs, service delivery), health extension workers spend more time on capacity building part for model HHs and they gave extra training, support and follow up to those who were selected to be role models. This training, support and follow up may bring knowledge and skill development to the model and it made them practice health packages well compared to non model HHs [3]. Findings from previous studies [8,9] revealed that, model families have good utilization of maternal health services. Mothers utilizing those services are more likely to gain access to other preventive services which commonly target children under five year of age. In addition to that, national workshop on maternal and new born health recommend that, the package for model families should include essential indicators of maternal and newborn care to advance maternal and neonatal health at the community level. This may indicate that they consider model households to have advantages compared to non model households [11].

About 99(30%) of model HHs have three or more rooms compared to 65(20%) in non model HHs, which is a significant difference between the two groups. Model households are likely to improve their housing conditions. According to a study conducted in Keffa Sheka, south west Ethiopia [12], fewer number of rooms was a risk factor associated with under five diarrhea. This may be due to the fact that when there is overcrowding in the HH, the chances for contamination of water and food would be high.

Other important improvements in household condition are having a separate sleeping place for domestic animals and having separate kitchen. About 74(27%) and 37(15%) did not live with animals in the same house and 88(27%) and 51(16%) had a separate kitchen in model and non model HHs respectively showing significant difference that indicates a possible effect of model HH training. During the training period, health extension workers made follow up, provided supportive supervision and given health education might be attributed to the behavior changes being observed after training. A study conducted in Guinea Bissau indicated that, having domestic animals in the house is a risk factor for diarrhea in children [13]. Living with animals in the same house increases unhygienic condition of the HH and probability of getting zoonotic diseases [14].

This study found that 26(8%) and 86(27%) have no latrine to use, and feces were seen around the house in 57(17%) and 91(28%) model and non model HHs respectively, again showing possible effect of the training towards lowering under five diarrhea. Availability of latrine reduces diarrhea [15] and unclean environment associated with under five diarrhea [16]. Availability of latrine reduces fecal contamination in the domestic environment and, in turn, this prevents transmission of disease-causing organisms to human beings. This is also true for unclean environment, which can be good media for pathogens [15,16].

In this study a statistical difference in practicing hand washing after toilet use was observed.

In 106(32%) model HHs and 145(45%) non model HHs care takers didn’t wash their hands after toilet visit. This difference may be due to model HH training. One study conducted in Pakistan indicated that hand washing after toilet visit significantly decreases incidence of diarrhea in under five children [17].

The study found good coverage of vitamin A supplementation, introduction of supplementary feeding in the age between 6-9 months and exclusive breast feeding in both model and non model HHs with no statistically significant difference between the two groups. This may be due to the expansion of health extension program [4-10]. Hence these programs are noted to play a great role in the prevention of under five diarrhea [18,19].

This study has its limitations and strengths. Limitations include; the information on the prevalence may not reflect the actual situation that may be observed in the various seasons of the year which could be addressed by longitudinal study by other researchers. Moreover, the absence of clear demarcation between model and non model with reference to distance (closeness of model and non model) may have created information contamination as well as diarrheal disease transmission to the model HH members and vice versa. The effect of food hygiene was not assessed due to resource limitations. The strength of this study is that, data collectors were blinded regarding whether each household was model or non model inorder to reduce interviewer bias.

## Conclusion

Diarrhea among under five children significantly reduced among families who fully implemented basic health packages. Since the finding suggests that being a model HH can have a positive impact on diarrhea morbidity among under five children, the model household training need to be scaled up in order to decrease under five diarrhea in the community. Furthermore attention should be given to water handling practice and information on how to care during maternal morbidity.

## Competing interests

The authors declare that they have no competing interest.

## Authors’ contributions

FB and YB participated from the conception to the final write up of the study. Both authors read and approved the final manuscript.

## Authors’ information

FB has Masters in Public Health and staff of Hawassa College of Medicine and Health Science, Ethiopia. YB is a senior Professor in Epidemiology and Public Health at Addis Continental Institute of Public Health, Ethiopia.

## Pre-publication history

The pre-publication history for this paper can be accessed here:

http://www.biomedcentral.com/1471-2458/14/187/prepub
